# Healthcare system barriers to long-term follow-up for adult survivors of childhood cancer in British Columbia, Canada: a qualitative study

**DOI:** 10.1007/s11764-017-0667-3

**Published:** 2017-12-08

**Authors:** A. Fuchsia Howard, Arminee Kazanjian, Sheila Pritchard, Rob Olson, Haroon Hasan, Kelly Newton, Karen Goddard

**Affiliations:** 1 School of Nursing, The University of British Columbia, T201 – 2211 Wesbrook Mall, Vancouver, Canada; 2 School of Population and Public Health, The University of British Columbia, Vancouver, Canada; 3 Division of Hematology/Oncology, BC Children’s Hospital, Vancouver, Canada; 4 Department of Surgery, The University of British Columbia, Vancouver, Canada; 5 Radiation Oncology, BC Cancer Agency – Centre for the North, Prince George, Canada; 6 Department of Radiation Oncology, BC Cancer Agency, Vancouver, Canada

**Keywords:** Survivorship, Childhood cancer survivor, Oncology, Qualitative, Healthcare system, Health services

## Abstract

**Purpose:**

Risk-stratified life-long follow-up care is recommended for adult childhood cancer survivors (CCS) to ensure appropriate prevention, screening, and management of late effects. The identification of barriers to long-term follow-up (LTFU), particularly in varying healthcare service contexts, is essential to develop and refine services that are responsive to survivor needs. We aimed to explore CCS and healthcare professionals (HCP) perspectives of healthcare system factors that function as barriers to LTFU in British Columbia, Canada.

**Methods:**

We analyzed data from 43 in-depth interviews, 30 with CCS and 13 with HCP, using qualitative thematic analysis and constant comparative methods.

**Results:**

Barriers to accessible, comprehensive, quality LTFU were associated with the following: (1) the difficult and abrupt transition from pediatric to adult health services, (2) inconvenient and under-resourced health services, (3) shifting patient-HCP relationships, (4) family doctor inadequate experience with late effects management, and (5) overdue and insufficient late effects communication with CCS.

**Conclusions:**

Structural, informational, and interpersonal/relational healthcare system factors often prevent CCS from initially accessing LTFU after discharge from pediatric oncology programs as well as adversely affecting engagement in ongoing screening, surveillance, and management of late effects.

**Implications for Cancer Survivors:**

Understanding the issues faced by adult CCS will provide insight necessary to developing patient-centered healthcare solutions that are key to accessible, acceptable, appropriate, and effective healthcare.

## Background

Cancer survivorship is now widely considered a distinct phase of cancer care that warrants specific efforts to deliver appropriate care. Moreover, this care must address the unique and complex needs of specific cancer survivor populations. The overall cure rate for all childhood cancers now exceeds 80% [1], leading to growing numbers of survivors who require long-term follow-up (LTFU) throughout adulthood. Many of these childhood cancer survivors (CCS) will have significant long-term health risks and experience late effects resulting from their previous therapies. By age 45, CCS have an estimated cumulative prevalence of any chronic health condition of 95%, and for a disabling, life-threatening chronic condition, 80% [2, 3]. The risk of treatment-related late effects, such as pulmonary dysfunction, endocrinopathy, cardiovascular disease, and anxiety, rises substantially with age [2–5]. Furthermore, psychological late effects are extremely common, as numerous CCS experience anxiety, fear, depression, and posttraumatic stress syndrome [6–11]. Leading North American and European organizations now recommend that all CCS receive risk-stratified, lifelong follow-up care to ensure appropriate prevention, screening, and management of late effects [12–15].

There is no conclusive evidence of the optimal healthcare services model for LTFU for CCS, and various models of care and different permutations of services have and will continue to be established. LTFU programs are available in some cancer centers in the USA [16, 17], Europe [18], and Canada [19, 20], yet it is widely recognized that most CCS are accessing care in the community with adult healthcare professionals (HCP) who are likely to be unfamiliar with survivor follow-up recommendations [21–23]. Additional barriers to comprehensive CCS care include limited capacity for survivor care within cancer treating institutions and poor communication between cancer centers and primary HCPs [10, 20, 24–27]. Survivor’s insufficient knowledge of their diagnosis, treatment, and potential consequences is also a well-recognized barrier to LTFU [24, 27–30]. Further barriers are likely influenced by the larger healthcare system within which this care is situated, that is the resources, individuals, and institutions related to the financing, regulation, and provision of health actions/services [17, 31]. In short, participation in late effects prevention, screening, and management is dependent on the healthcare available to CCS. Understanding the healthcare system factors that CCS experience as barriers to quality care is necessary to develop best practices and healthcare services that are responsive to patient needs, that is, patient-centered.

Incorporating patient perspectives into healthcare service planning is increasingly recognized as key to delivering accessible, acceptable, appropriate, and effective healthcare [32–34]. Survivors, themselves have firsthand knowledge of the ways in which healthcare services meet their needs and the essential components that can facilitate or prevent their engagement with services. Yet, limited research has focused specifically on survivor’s experiences of follow-up services [34, 35]. HCP working with CCS also witness the difficulties their patients encounter and are adept at assessing challenges arising in the healthcare context within which they work. Thus, our aim in this study was to examine CCS and HCP perspectives of healthcare system factors that function as barriers to LTFU in the province of British Columbia, Canada. In Canada, healthcare is delivered through a publicly funded healthcare system wherein the provinces are required to provide universal coverage for medically necessary hospital and physician services. This research presents an opportunity to examine barriers to CCS LTFU in the context of a publicly funded healthcare system.

## Methods

This paper builds on prior analyses of 30 in-depth interviews with CCS wherein we described survivor’s experiences of social isolation [36] and the ways in which survivors manage their medical and psychosocial challenges [37]. For this study, we also conducted interviews with 13 HCP involved in the LTFU of CCS and then analyzed all 43 interviews using qualitative thematic analysis and constant comparative methods. Our study was granted ethics approval through the joint University of British Columbia and BC Cancer Agency Research Ethics Board.

### Guiding theory

We applied the theoretical lens of relational autonomy to this research, a detailed description of which has been previously published [37]. In brief, a relational autonomy lens enabled us to contextualize survivors’ experiences by framing the research and interview questions so as to explore the personal factors, interpersonal relationships, and wider social contexts that shaped the experiences of the CCS [38, 39]. For example, we asked study participants to reflect on how HCP, healthcare services or programs, and healthcare organizations helped and/or hindered them in managing their physical and emotional issues. During data analysis, we continued to apply a relational autonomy lens to consider how CCS were situated in the larger healthcare system and how aspects of this system complicated LTFU.

### Setting and study participants

At the time of this research, specific CCS care was available in British Columbia to survivors 18 years of age and younger at the BC Children’s Hospital. For adult survivors of childhood cancer, there existed one unfunded clinic at the BC Cancer Agency, through which approximately 450 survivors were followed. The remainder of CCS received their healthcare through a general practitioner in their community, provided they had a primary care provider.

We recruited CCS in British Columbia using convenience sampling of survivors who were diagnosed with cancer prior to 19 years of age and between 19 and 45 years of age at the time of the study. We distributed study fliers to potential participants who received LTFU at BC Children’s Hospital or BC Cancer Agency clinics, and posted study information on online forums and websites. We interviewed all CCS who contacted the research team and then used purposive sampling to recruit participants with diverse characteristics, including age at diagnosis, current age, diagnosis, and rural and urban residence, until we reached data saturation. Thirty CCS participated in this study, 25 of who were recruited through a clinic and 5 online. The CCS received their initial cancer diagnosis 9 to 38 years previously (mean 22 years ago), with 3 survivors treated between 1970 and 1979, 13 treated between 1980 and 1989, 13 treated between 1990 and 1999, and 1 treated in 2002. These CCS ranged from 19 to 43 years of age at the time of interview. See Table 1 for further demographic information, disease characteristics, and late effects.

**Table 1:** Participant self-reported demographic information, disease characteristics, and late effects

Demographic characteristics	All *n* = 30 (%)
Age	
20–24	16
25–29	27
30–34	30
35+	27
Gender	
Male	40
Female	60
Place of residency	
Greater Vancouver area	70
Other	30
Marital status	
Single	73
Married	27
Living arrangement	
Alone	30
With roommates	13
With a partner/spouse	27
With parents	30
Level of education	
Did not complete high school	7
Completed high school	23
Completed university/college	70
Employment status	
Unemployed	13
Student	10
Employed part- or full-time	77
Disease characteristics	
Age at first diagnosis	
0–4	27
5–9	33
10+	40
Treatment era	
1970–1979	10
1980–1989	43
1990–1999	43
2000+	3
Type of cancer	
Leukemia and lymphoma	53
Brain tumor	20
Sarcoma (not including brain)	20
Other solid tumors	7
Treatments	
Radiation therapy	90
Chemotherapy	97
Surgery	37
Bone marrow transplant	3
Late effects and health problems	
Impaired growth and development	43
Bone, joint, or soft tissue late effects	40
Anxiety or depression	37
Second cancer	30
Learning difficulties or cognitive impairment	30
Impaired sexual development or infertility	30
Endocrine late effects	30
Hearing impairment	27
Visual impairment	23
Digestive late effects	20
Respiratory late effects	17
Cardiovascular late effects	17
Dental late effects	13

Percentages might not add to 100% because of rounding

We identified HCP involved in the provision of LTFU in collaboration with program directors at the provincial BC Cancer Agency and the BC Children’s Hospital. We also used snowball sampling whereby we asked HCP who participated in the study to identify other HCP involved in LTFU. Because LTFU is largely delivered by a small number of providers in British Columbia, program directors and the individuals we interviewed were familiar with other HCP involved in CCS care, with the exception of primary care providers. We recruited HCP via email and telephone and interviewed 13 of the 17 HCP identified, which included 6 physicians (2 pediatric oncologists, 1 adult oncologist, 1 neuropsychiatrist, 1 cardiologist, and 1 family physician), 2 registered nurses, 1 social worker, 1 counselor, 1 patient/parent advocate, and 2 healthcare administrators. To maintain participant confidentiality, we have not included a further breakdown of HCP demographics because this could make it possible to identify participants.

### Data collection procedures

One investigator conducted in-depth interviews with CCS, while two investigators conducted interviews with HCP. All interviews were conducted between April 2011 and April 2012, with 30 interviews conducted in-person (20 CCS and 10 HCP) and 13 conducted via telephone (10 CCS and 3 HCP), to accommodate participant preference, availability, and location of residence (living outside of the city center). The interviews lasted between 45 and 120 min. We digitally recorded and transcribed verbatim all interviews. We used an interview guide to explore common themes and topics across interviews, and we asked participants open-ended questions to garner their perspectives. We refined our interview questions as we proceeded with analysis and interviews as a means of exploring important ideas and emergent findings.

### Data analysis procedures

We analyzed the CCS and HCP interview data using inductive, thematic analysis, and constant comparative methods [40, 41]. Two investigators read the interview transcripts numerous times and developed an initial coding frame of the important concepts, events, interpretations, and experiences related to the ways in which CCS manage their medical and psychosocial challenges. We used the qualitative data management software program NVivo^™^, to apply the coding frame to all interview data. One of the broad themes arising from this inductive coding was CCS interactions with health and social services, which included positive and challenging experiences with programs, services, institutions, and various HCP. We then retrieved all transcript data from this broad theme and two investigators coded this data, again using an inductive approach, to identify important themes that recurred within the interviews and were evident in multiple participants’ accounts, including both the CCS and HCP transcripts. We paid particular attention to identifying healthcare system factors, specifically HCP, resources, and institutions, that were described as barriers to cancer-related follow-up. We then compared and contrasted emergent themes until we were confident that we had captured the predominant ideas, interpretations, and perspectives evident in the participants’ interviews. As a research team, we then refined these themes for clarity and completeness.

## Results

The CCS and the HCP considered LTFU to be critical to the health and wellbeing of survivors. Yet, five themes highlighted CCS and HCP perspectives of healthcare system factors that functioned as barriers to accessing comprehensive, quality LTFU. These barriers were associated with the following: (1) the difficult and abrupt transition from pediatric to adult health services, (2) inconvenient and under-resourced healthcare services, (3) shifting patient-HCP relationships, (4) family doctor inadequate experience with late effects management, and (5) overdue and insufficient late effects communication with CCS (see Table 2).

**Table 2:** Healthcare system barriers to long-term follow-up

Main theme	CCS perspectives	HCP perspectives
The difficult and abrupt transition from pediatric to adult health services	• “Kicked out” of pediatric services • Left to navigate adult services without preparation • Worried they were not receiving healthcare	• No professional ownership over transition • Problematic timing coinciding with developmental transition • CCS continued reliance on parents
Inconvenient and under-resourced health services		
Location of multiple services	• Burden associated with travel and taking time off work • Burden amplified for CCS with a disability and living far from services	• Financial burden resulted in missed appointments and eventual loss to follow-up
Lack of HCP time	• Family doctors unable to address numerous health challenges • HCPs already caring for an unmanageable number of patients	• Quality of care compromised • No time to focus on medium- or low-risk survivors
Limited designated LTFU funding		• Insufficient dedicated resources for survivorship • Survivorship not the priority
Shifting patient-HCP relationships	• Lack of trusting patient-HCP relationship (wherein CCS felt “known”) did not engender trust in care • CCS were reluctant to seek care and discuss late effects with HCP when there was not a trusting relationship	• Adult HCP lack the time necessary to build these positive patient-HCP relationships, essential to quality care
Family doctor inadequate experience with late effects	• Inadequate knowledge of cancer treatment details and health risks • Negative experiences lead CCS to seek out oncology HCP	• The consequence of recent and evolving evidence about late effects and small numbers of CCS followed by family doctors
Overdue and insufficient late effects communication with CCS	• Prevented CCS from engaging in late effects prevention, early detection and obtaining support • Prevented CCS from incorporating information into life decisions	• Late effects discussions difficult for CCS • Failure to communicate information all along the health care continuum • Cohort of CCS currently lost to follow-up

### The difficult and abrupt transition from pediatric to adult healthcare services

All of the CCS in this study had transitioned out of pediatric services and were followed by a general practitioner, a specialist, or through a shared care model involving a family doctor and specialists. The majority of CCS experienced an abrupt change in HCP, rather than a gradual process of transition. They felt “kicked out” of the children’s health services, contributing to feelings of abandonment, neglect, and frustration. A number of the CCS perceived that they were left to navigate a confusing adult system without sufficient preparation or support. They were unsure of where to turn for help and worried about whether or not their healthcare needs were being looked after. When follow-up appointments and late effects screening became less frequent, as was often the case with a survivor’s transition from pediatric to adult services and in line with clinical guidelines, CCS became anxious and nervous that they were not receiving the necessary care.

It [post-treatment follow-up appointments] went from every three months to every six months and then every year. And then all of a sudden here’s a letter saying, okay, well we don’t want you to come in here anymore and your options are; you can do letter follow up, you can come into the [hospital] once a year... I felt it was a little uncaring. They say oh we can do a letter follow up and basically the letter follow up was saying well if you have any concerns we can talk about it… I mean I could have been lost in the paper shuffle. [39-year-old, ALL survivor]

HCP’s described the transition from pediatric to adult health services as historically problematic because of no oversight of the transition process and no centralized ownership or responsibility for guiding survivors over time. HCP attributed the limited professional guidance available to survivors to the organization of health services wherein no particular professional was primarily responsible for, nor tasked with, managing and truly facilitating the transition of CCS.

What we’re really doing is transferring their care, we’re not actually transitioning them through the steps of educating them to take responsibility for their healthcare, and we’re just passing the information along. And there really has to be some centre or repository so that people have the information, you know, who do you call and where do you go look and who should you see and what the surveillance should be. But that doesn’t exist. [HCP]

The timing of this healthcare transition also coincided with the developmental transition from adolescence to adulthood, which, according to HCP, was in itself complicated by late effects. This was especially a troublesome for CCS who experienced psychosocial difficulties or neurocognitive impairment, most prominently brain cancer survivors. HCP perceived that the support provided to children and adolescents failed to adequately prepare survivors with the life skills necessary to navigate the adult world. As such, HCP perceived those who were struggling to be independent, high functioning adults, to be overwhelmed by the numerous different transitions they were expected to manage simultaneously, to work, to postsecondary education, to financial independence, and to emotional and social independence. These struggles compounded the difficulties CCS faced as they transitioned to the adult healthcare system. Ultimately, survivors often continued to be reliant on their parent’s involvement in their care and did not learn how to navigate the adult healthcare system until they were much older, if at all. If parental or HCP support dissipated, some CCS were unable to access services or effectively engage with HCP.

Eventually, the parent maybe steps out, they’re kind of left with no one. So most of them don’t really understand how to sort of be an adult patient in an adult care facility. Where they are really expected to show up for the appointment, make their own appointments, keep track of their pills, ask for new prescriptions because the adult healthcare is very much focused on the patient is responsible for all their own, making of all of those things whereas in the pediatric world it’s sort of taken over either by family or some other healthcare provider will sort of guide them through that sort of thing. Whereas an adult if you don’t show up for your appointment you might get one reminder but after that nothing so there’s not a lot of follow up so I think a lot get lost to follow up. [HCP]

### Inconvenient and under-resourced healthcare services

The CCS and the HCP described the healthcare services in British Columbia as inconvenient and at times, inaccessible, because of the location of multiple services. HCP lack of time to provide comprehensive quality care and limited designated LTFU funding created notable challenges that appeared magnified when contrasted with oncology treatment services.

#### Location of multiple services

The CCS had become accustomed to obtaining all of their healthcare in one place in the pediatric system. This benefit was not offered to adult CCS who were frequently followed by different and often multiple specialists. These specialists were concentrated in one large urban region of the province but spread throughout this region. In addition, survivors attended appointments with primary HCP, psychologists, nurses, counselors, and social workers who were spread throughout British Columbia. This decentralization of services resulted in a notable burden associated with travel itself, as well as taking the time required to travel to multiple medical appointments and obtain late effects screening (i.e., laboratory tests, CT scans, ultrasounds, MRI). Eleven of the 30 survivors indicated that “just getting to appointments” was a barrier to accessing care.

It’s kind of like a distance because when you went to [pediatric center] you had everything there and now since I left everything is like [adult health center], [hospital], [specialist], different areas. Now you have to fly around… it’s not all in one place. [24-year-old, ALL survivor]

This was even more difficult for the CCS who did not have a driver’s license because of a disability. They were dependent on family members or public transportation to attend multiple appointments scattered throughout the region. For example, the above participant further described that she relied on the bus despite feeling dizzy, clumsy, and disoriented because of challenges with hearing, visual, and neurocognitive impairment.

The burden of obtaining follow-up care was amplified for survivors who lived in rural areas and great distances from the large urban setting. CCS and family members spent time negotiating with employers to take time off work, making complex arrangements to manage their household, and arranging and paying for childcare, thereby assuming substantial related expenses.

My husband has to take time off work, I mean things that are in [small city] I can go by myself and I used to do that until we had our son and now things are complicated because I have to truck him around too. We don’t have childcare, we can’t afford it and there really isn’t a heck of a lot of it available…. He has to take the time off either to stay here with [son] or to come with. We have to drive forty-five minutes to [small city] for things like when I have ultrasounds even and CAT scans that sort of thing. My appointments to see my obstetrician, my oncologist, any other specialist that may be around. And then we have to go to [city] which is about two and a half hours away for echocardiograms, and things like MRI’s we have to go all the way down to [large city] which is about an eight or nine hour drive. [26-year-old, ALL survivor]

HCP also acknowledged the out of pocket costs of care assumed by CCS who had to travel great distances. HCP suggested that as time went on they simply lost touch with survivors:

It’s expensive to come to Vancouver. They have to pay for travel and accommodation themselves so often times if they feel well they decide I’m not goanna come this year. If it’s five, six hundred dollars for a plane ticket to get down and I don’t feel bad, I’ll just cancel my appointment. And then they forget to make it the next year and eventually they just get lost in the system and that sort of thing. [HCP]

#### Lack of HCP time

Seven of the 30 CCS perceived that HCP lacked sufficient time to adequately address the numerous challenges survivors experienced. The CCS considered family doctors to be particularly overburdened and unable to create time in their busy workday to fully understand and address their health issues. This shortage of time was not regarded to be the fault of the practitioner. Rather, the healthcare system as a whole was described as lacking sufficient physician specialists and family doctors, resulting in each practitioner being responsible for an unmanageable number of patients. As such, CCS perceived that HCP were forced to compromise the quality of care they provided to be able to manage their workload.

Five of the 13 HCP also considered HCP time pressures to be a barrier to the provision of adequate care. Lacking time, the HCPs (specialist physicians, social workers, nurses, psychologists, and counselors) limited the aspects of care they focused on and prioritized their complex and acutely ill patients, leaving little time to spend addressing CCS’ chronic needs. Other HCP described unsuccessfully attempting to do the work of two professionals, thereby compromising the quality of care they could provide.

There was this huge, huge gap where the transitions weren’t really, we weren’t able to do the best job we could because there was only one follow-up nurse. So I kind of took that under my wing as well, I kind of do the two things both. I don’t have enough time for either one really to do a good job. . . So its very time consuming, there’s definitely two jobs there. So I’m skimming the surface of both. [HCP]

The HCP frequently made reference to “huge wait lists.” For example, the few psychologists familiar with the needs of CCS had wait lists of up to 6 months and were only available for a limited number of short appointments, yet survivors were frequently in need of ongoing counseling. Lack of time also meant that many HCP did not accept CCS as new patients. General practitioners could not add a complex CCS to their already busy medical practice, and as such, pediatric specialists frequently continued to care for adult CCS at high risk for late effects. However, specialists feared that because they only had time to focus on high-risk survivors, the medium- and low-risk survivors “slipped through the cracks.”

One of the biggest barriers is the fact that we don’t have anywhere to send these kids. So a lot of them, yes, can be seen by a GP but a lot of them need more than that but they can’t go to [specialists]… It’s the in-between ones, the ones that you can’t just send them to a GP and hope that they’ll learn about late effects. You know there are some things that you want to make sure they get done so you really try and teach the patients that for sure. [HCP]

#### Limited designated LTFU funding

The CCS did not discuss healthcare service funding challenges; however, 10 of 13 HCP described LTFU funding as inadequate to support the needs of the CCS population in the province. The HCP saw the priority of the adult cancer centers to be the provision of adult cancer treatment, while the priority of pediatric cancer centers to be pediatric cancer treatment. While “lip service” had been paid to survivorship care, neither had sufficient dedicated resources nor a formal structure to assume the LTFU of adult CCS. The surveillance and follow-up program cobbled together by clinicians was described as subpar and insufficient, leaving HCP’s to work with few resources and minimal time to provide satisfactory, “practical” care for extremely large patient loads. The HCP believed that funding was overlooked for this population because the decision-makers had limited knowledge of the severity and prevalence of late effects and the lifelong nature of these risks.

I really don’t think that the majority of people understand that a lot of these kids and then adults are living with a disability. The consequences of that is a lack of funding. That’s probably the big one, that we don’t plan for a three year old to have seventy-five years of disability. [HCP]

### Shifting patient-healthcare provider relationships

For the most part, the CCS had interacted with numerous HCP over time, and they considered the formation of a positive patient-provider relationship essential to LTFU. The quality of these relationships, based on shared experiences, feeling “known” and survivor’s perceived trust, influenced the nature of the survivorship care they received, yet many of these relationships shifted over time. The “close” relationships that CCS had with pediatric HCP who had been involved in their cancer treatment were highly valued, “irreplaceable,” and as one survivors stated, “you don’t really feel that you filled that childhood relationship again with anybody.” [30-year-old, Hodgkin Lymphoma survivor]. The CCS felt cared about because they perceived that providers put in great effort to “get to know me as a person.” However, this degree of caring was rare in subsequent relationships with adult HCP.

The Children’s Hospital was, how do you describe it? Like everything was geared toward a kid and everyone cared about you and things are decorated and, it’s geared towards kids and you’re happy. And then the [adult center] it’s, it’s an adult world. Not that it’s bad but it was just different like going from high school to university almost. … You’ll be more of a number whereas in high school they, they know more about you and they care more about you. I don’t want to say that they care more about you but it feels like that. [38-year-old, ALL survivor]

A number of the CCS also highly valued relationships with adult HCP who were in an adult oncology setting. They depended on these individuals as their primary source of health-related information and support. They trusted these HCP, which translated into trust in the larger healthcare system, confidence in their LTFU care, and reassurance that they were getting the appropriate care.

I’ve relied on my oncologist to look after everything and to follow up. I just had an MRI actually yesterday and she, she had ordered it and knowing that she’s following up on me makes me sure that later on I’m not going to, I mean I’m trying to avoid any possible things of cardiac, anything happening to the heart and with the thyroid and everything. She’s always checking up on me and making sure that, I trust that, that she’s able to look after me and I trust the system that I’m in right now. [32-year-old, Rhabdomyosarcoma survivor]

Trusting relationships also enabled CCS to feel comfortable raising their concerns, asking questions, and requesting additional support. The CCS described making concerted efforts to attend their medical appointments for fear that their oncology HCP would notice their absence. Despite valuing their relationship with their oncology HCP, CCS felt they were a “burden” and “guilty” using acute oncology services when they were healthy, and uncomfortable attending appointments at an adult treatment facility where there were “old sick people.”

HCP also highlighted the importance of fostering a positive patient-provider relationship with survivors by “knowing” their patients, as a means of providing quality care. Oncology HCP were particularly adept at fostering meaningful relationships with CCS patients because they had, “treated these patients for however many months or years, and they know them and their families really, really well, and they usually trust us. So they’ll talk about things to us that they perhaps wouldn’t talk about to other specialists” [HCP]. However, HCP saw adult providers as lacking the necessary time to build these relationships.

The survivors stressed the importance of having quality relationships with HCP outside of cancer care, particularly primary HCP. Many of the CCS who had a family doctor described them as willing and able to address “regular health” problems. However, most CCS described the quality of the patient-provider relationship they had with their family doctor as impersonal, in part because they were not present at the time of treatment, but also because of the limited effort family doctors were seen to put into getting to know them. The lack of a strong patient-provider relationship did not engender trust in this care, with CCS expressing reluctance to even visit their family doctor, let alone to discuss cancer-related worries, describe late effects symptoms, or follow medical advice.

### Family doctor inadequate experience with late effects management

Both the CCS and the HCP expressed concern that overall, family doctors possess inadequate knowledge of the cancer history of individual patients as well as late effects screening and management guidelines, to provide comprehensive LTFU. The majority of the CCS reported that their family doctor was aware of their cancer history but did not know the diagnosis or treatment details. For some, discussions with their family doctor were limited to being asked minimal brief questions, while others had never had a conversation about their cancer.

At some point, driven by worry and suspicion that there would be consequences of having survived cancer, or by the onset of illness or health issues (e.g., fatigue, weight gain), many of the CCS sought out cancer-related advice or care from a family doctor. However, this experience was not always positive. Some CCS described the family doctor as “apprehensive” and “avoidant” of cancer-related discussions. Other CCS interpreted family doctors’ responses to their questions about late effects screening as defensive. The CCS felt they had to justify their worries and their questions and were placed in the “awkward” position of attempting to educate their family doctor. Moreover, CCS often felt they had to be proactive in requesting specific late effects screening and monitoring (i.e., breast examination, mammogram, thyroid ultrasound, blood tests) because their general practitioner rarely instigated investigations. A few of the CCS were “laughed at” or their concerns dismissed, leading to frustration and loss of faith in their general practitioners’ willingness and ability to provide care.

You go in and you tell them [general practitioner] you’ve got these issues and they go well, maybe it’s something you’re eating or you should stop eating that. And you’re like, okay, but there’s an issue why it’s happening, right? And, so they go well, it could be this or well maybe it could be that? And I said well, I have had cancer before and I’m kind of worried it might be not that it’s the same kind of cancer but maybe it’s something else, who knows? And they’re going well, the likelihood of that is not there and they don’t take it into consideration. And I find like you get stalemated... And then they stop at that, they never do a blood test, they never check it out, they never go any further than that. [31-year-old, ALL survivor]

The CCS who had negative experiences learned not to rely on their primary HCP, and 23 of the 30 CCS reported that they make concerted efforts to seek out cancer-related care from an oncology HCP rather than their primary HCP. When a specialist successfully communicated the survivors’ risks and recommendations for risk-based care to the family doctor, their knowledge and willingness to be involved improved.

Dr. [specialist] was talking and sharing test results and reports back with my GP and I think she [general practitioner] finally understood what my needs were. So now she’s a little bit more on the ball with, okay, well we’ll have to get you in for a mammogram, we’ll have to go test you for your bone scans. Now we’ve got to get this now and I think she’s a little bit more aware that it may be different than say any other patient. [26 year-old ALL survivor]

The HCPs discussed primary HCP inadequate knowledge as problematic, but this was considered to be a consequence of relatively recent and evolving evidence about late effects that would only very rarely be applicable in their practice and poor collaboration efforts between specialist and primary care providers. According to HCPs involved in LTFU, family doctors are not the only HCP lacking knowledge of the health risks facing CCS, which places the onus on specialists to share their expertise.

I think we need to do more outreach in the community…. Involving family practitioners in their care, making sure they know their part in the team as well, and letting them know about some of these late effects. The other thing you find out is that many family practitioners have no idea, like many of my colleagues don’t even know. I was asked recently by one of my adult colleagues who treats brain tumors in adults, he treated somebody with radiation to their spine and brain and he was going to discharge them and was like, well did you treat their spine, well what about the thyroid, what about the risks of under activity, you need to screen for thyroid cancer. [HCP]

Family doctors were perceived to be receptive to acquiring the requisite knowledge, keen to participate in the provision of LTFU, and “grateful to get the information.” Once these collegial relationships had been established, family doctors often sought out advice, especially when managing CCS with complex health needs, and also assumed responsibility for LTFU. However, this was complicated by evolving guidelines and inconsistent risk-based screening recommendations. Moreover, family doctors were not always able to order specific tests (i.e., MRI, echocardiogram) that are usually ordered by specialists. All HCP agreed that the status quo restricted primary care involvement in LTFU and limited CCS access to comprehensive, risk-based care.

### Overdue and insufficient late effects communication with CCS

CCS and HCP described the knowledge survivors possess of their cancer treatments and late effects risks to be inadequate. The majority of CCS (22 out of 30) stressed the importance of knowing their health risks, yet they lacked sufficient details and this information was conveyed “much too late,” often after the age of 18 years or when late effects developed. Receiving this information was “nerve wracking” and “distressing.” The CCS also described missing the opportunity to “look after myself” by engaging in preventive behaviors or early detection and treatment.

I didn’t really understand the risks I just suddenly had all the problems. And it was a bit of a shock because I didn’t really feel I had been warned. I think I should have been told about the risk of skin cancer because I think I would have gone to see someone sooner. I waited two years before I actually mentioned it to anybody so I think I should have been warned about that. And when I spoke to the surgeon he said, yes, we’re seeing this a lot in, in former patients who had cranial radiation. It sounded like it was a common problem and I think I should have been told to look out for that because I always felt I was too young for skin cancer. [34-year-old, ALL survivor]

Furthermore, CCS would have factored their health risks into their life decisions (i.e., moving away from parents, seeking out a relationship, getting married, and having children) had they known earlier. This was particularly important for women now facing possible infertility.

The issue that has caused me most concern because I don’t think I was as aware of it and didn’t feel like it was as monitored would have been the fertility issues. And I think maybe because I was a teenager at the time those long term effects weren’t necessarily discussed with me. So I don’t think I appreciated the extent of how limited my window might be for having a child compared to other women. I don’t think I appreciated the fact that I would likely go through menopause a lot earlier. And I think that, that has caused me more concern because I felt like I wasn’t as aware of it. [32-year-old, Hodgkin Lymphoma survivor]

Inadequate knowledge of the specifics of their cancer treatment and late effects risks also prevented CCS from seeking out appropriate medical and emotional support and resources, such as peer support, professional psychosocial support, and online information and education.

HCP involved in LTFU described the anger and frustration CCS experience when informed of their late effects risks so long after treatment. CCS were given false hope that they were “cured” and then later met the disheartening realization that they would likely suffer ongoing and at times debilitating health issues. These HCP framed this as a gap in patient care, with 9 out of the 13 HCP remarking on the failure of many other community HCP across the care continuum to initiate formal discussions with survivors. These were difficult discussions because they brought back highly charged emotions for survivors. However, in the absence of these discussions, CCS often chose not to maintain consistent follow-up.

They’re (CCS) not aware of the problem, the fact that they may develop a problem and it’s not going to be for ten years or fifteen years down the line. And so they’re told oh you’re fine you don’t ever have to see me again . . . They don’t come back, no appointment is made and so then it’s not until they actually get very sick that somebody recognizes oh, they maybe should have been followed. [HCP]

HCPs involved in LTFU described the evolution of efforts to educate CCS, such that currently, specific and formal information about cancer treatments, late effects risks and recommendations for prevention, screening and LTFU are discussed with survivors. Yet, HCP also acknowledged that a large cohort of survivors likely exists that is unaware of their risks and is currently not receiving follow-up care.

Just this afternoon I got a call from a patient that was treated at the age of about three, got cranial radiation, parents stopped treatment towards the end of his therapy and refused to ever come back for a follow-up. And so that patient had never had any information. We gave her a summary and went through things and sent it to her family doctor who was obviously very diligent doing all of these things. [HCP]

## Discussion

In this research, CCS and HCP involved in LTFU programs identified healthcare system barriers to LTFU in British Columbia, Canada, which included the difficult and abrupt transition from pediatric to adult health services, inconvenient and under-resourced health services, shifting patient-HCP relationships, primary HCP inadequate late effects experience, and overdue and insufficient late effects communication with CCS. Given the current state of the development of LTFU services in Canada and elsewhere, our results offer several important contributions to the literature.

Formal programs to prepare CCS to transition from pediatric to adult health services have been developed and implemented worldwide, including in British Columbia. Transition programs focus largely on educating survivors, considering that CCS lack of knowledge of their prior diagnosis and treatment is well documented [28]. This has spurred on the development of treatment summaries, survivorship care plans, and electronic patient portals to address survivor’s educational needs [42–45]. In fact, current guidelines recommend all pediatric cancer survivors receive a survivorship care plan [14]. However, recent research suggests that even when survivors are educated about their disease and treatment history, risk for late effects, and need for follow-up, they might be unclear of the expectations of ongoing survivorship care and how to advocate for themselves in adult medical settings [35]. Indeed, this was the case for participants in our study, and building on this, our findings suggest that survivors require pragmatic healthcare system information and coaching to assist them in accessing and obtaining the necessary adult health services. While instruction in self-management skills including proficiency using the adult healthcare system has been established as a necessary component for successful transition [46, 47], there are few resources to enable HCP to provide this type of support. Joint visits with providers from pediatric and adult providers prior to transition as well as peer transition navigators could potentially address this challenge and warrant further investigation. In line with current recommendations, our data also suggests that formal discussions about future health risks begin well before the transition out of the pediatric system, yet, it would perhaps be beneficial to have additional formal discussions on an ongoing basis, especially when survivors are not experiencing late effects. As argued by others [48], the ideal time to transfer a patient to a new provider might be when the patient is relatively clinically stable, rather than when they are facing the challenge of establishing provider-patient relationships under the stress of a new diagnosis. Indeed, trusting patient-provider relationships was considered essential to quality care and motivated CCS in our study to continue with LTFU.

In our research, CCS lack of knowledge contributed to missed opportunities to engage in preventive health behavior, obtain medical care, and access psychosocial support, and it is unknown whether improving survivor knowledge with treatment summaries, survivorship care plans, and electronic patient portals will in fact improve their engagement with LTFU, screening, and preventive health behaviors. Perhaps, interventions to improve CCS knowledge will be most effective when embedded appropriately in the context of care delivery such that healthcare system barriers to LTFU evident in this study are simultaneously addressed and access to quality survivorship care improved. A recent study of CCSs found that healthcare self-efficacy (confidence to manage one’s health and healthcare) was higher among those who attended a survivorship clinic and had a regular source of healthcare (both non-cancer and oncologist) and any type of health insurance [49]. Routine engagement with HCP and services likely creates opportunities to comprehend information and appreciate risks over time. However, even in the context of a structured LTFU program, adherence to recommended screening tests has been found to be suboptimal, particularly among CCS older than 18 years of age [50]. Investigating ways to enhance survivor knowledge and evaluate strategies for effective teaching and testing methods and models of care that engage survivors in health self-management and prevent survivor follow-up attrition should be the subject of future research.

Our finding that the physical location of services is important to survivors is not new. For example, CCS in Swiss research emphasized the necessity of providing care in convenient locations [51]. However, much of the literature has framed this simply as survivor preferences rather than recognize the tremendous influence that the geographic location of services plays in creating unaffordable, indirect costs, and thus inaccessible healthcare. This burden is likely compounded for many CCS when considering that CCS, and especially central nervous system tumor survivors, have an increased probability of unemployment and of receiving government financial assistance [52]. Indeed, in a recent Canadian study, CCS with a low socioeconomic status and those residing more than 50 km from a publicly funded specialized CCS clinic were less likely to access this care [53]. Thus, it is important for clinicians to discuss financial challenges with CCS and limit travel when possible. Innovative strategies to minimize the indirect costs associated with LTFU (e.g., travel, taking time off work, and arranging childcare) that will require future investigation and healthcare organizational investment could include tele-health, rotating clinics across the province, alternating in-person and remote appointments, electronic survivor care plans that can be accessed by survivors and primary care providers, and efforts to explicate and formalize shared models of care [54, 55].

Our results suggest that the relationships between CCS and LTFU HCP influences patient-provider communication and ongoing health seeking and engagement behavior that is essential to effective, quality care. In a recent survey of pediatric oncologists in the USA, 91% identified the most frequent barrier to transferring CCS to adult care to be perceived emotional attachment of the survivor to the provider [48]. Additional barriers to transfer included the provider’s attachment to the family/patient (85%) and parent’s attachment to the provider (90%). Among our participants, a trusting patient-provider relationship was often expressed as a positive emotional attachment, which was influenced by CCSs’ experiences of “feeling known” by their HCP. Our use of the relational autonomy theoretical lens in this research enabled us to focus on and highlight the complex and marked influence of key relationships to CCS health. This is in line with research by Thorne [56], who found that cancer patients’ experiences of “being known” in a meaningful way were largely shaped by patient-provider communication and were an essential component of human connection. Perhaps, fostering trusting patient-provider relationships through meaningful communication rather than framing emotional attachment as a barrier and patient-provider communication as simply information based, would not only improve patient satisfaction, but also improve LTFU attendance, adherence to screening recommendations, and ultimately, patient outcomes. Of importance, this would require support for HCPs to spend the time required to establish and maintain rapport and communicate effectively with CCS in all their clinical encounters. Communication skills training interventions for HCP caring for adult cancer survivors are under investigation [57] and would perhaps also be applicable in the context of the CCS population.

HCP limited knowledge of late effects risks and screening recommendations is well recognized [47] and has provided the impetus for promising information-based solutions, primarily in the form of survivorship care plans. The majority of adult primary HCPs in a study by Nathan and colleagues [22] were willing to care for CCS. However, in a more recent study [58], primary HCP who were sent survivorship care plans generally felt uncomfortable using the plan and providing survivorship care. Like Iyer et al. [58], our findings also suggest that provider educational efforts and documentation could perhaps be augmented if combined with explicit healthcare organizational efforts, care processes, and formal linkages between HCP, which aim to enhance collaborative practice. Clearly, this is an area worthy of further research.

Since the collection of the interview data in our study, the adult and pediatric cancer organizations in British Columbia created the Adult Childhood Cancer Survivors Program and the Late Effects Assessment and Follow-up (LEAF) clinic. This new program and clinic have dramatically improved the state of health services for CCS in the province and have addressed what we, and others, have found that limited resources, lack of HCP time, and survivorship funding were considerable barriers to the provision of LTFU [16, 59, 60]. Healthcare organizations worldwide have echoed the recommendations of the U.S. Institute of Medicine to develop and implement survivorship care; however, such organizations often have multiple competing service demands, particularly in the Canadian government-funded healthcare system context. Exploration of ways to promote optimal health outcomes in a time efficient, cost-effective manner is necessary to ensure CCS care is effective and sustainable [16].

Limitation of this study includes an overrepresentation of CCS who were already receiving cancer-related LTFU, either through a LTFU clinic, a family doctor or walk-in-clinics. As such, the healthcare system barriers that prevent CCS who are by and large disconnected from medical care were not represented in this research. Moreover, we had limited input from CCS whose LTFU was managed primarily by their family doctor. The missing from this study are the perspectives of CCS who have close, long-lasting relationships with their family doctor are satisfied with this ongoing follow-up. There is also a possibility that study respondents may have been more interested in CCS LTFU compared with the general population of CCS. Of note, our study sample included a large number of individuals at high risk for late effects, based on primary cancer treatment that included radiation therapy, compared to the population of CCS. Not surprisingly, a high percentage of these individuals also self-reported late effects, such as a second cancer. It is likely that these individuals have required specialist or oncology care for late effects that primary care providers do not manage, and could potentially perceive primary care providers as lacking knowledge when in fact this knowledge and late effects management are outside their scope of practice. Caution must be exercised when determining the relevance of study findings to other CCS populations and settings, especially with different healthcare system delivery structures, or CCS who may or may not have access to similar LTFU services. Nevertheless, reporting these findings in a Canadian context provides much needed locale-specific insights about CCS healthcare services that are also relevant nationally and internationally. Only one primary HCP participated in this study, despite significant recruitment efforts, and their perspectives are likely to be under-represented.

In conclusion, the CCS and HCP in this study identified specific structural, informational, and interpersonal/relational healthcare system barrier to initially accessing LTFU following discharge from pediatric care as well as continuing to engage in ongoing screening, surveillance, and management of late effects. Though specific to CCS, the study findings are likely germane to other young adults with a chronic illness, who transition from pediatric to adult services and require ongoing healthcare, such as individuals with type 1 diabetes, juvenile-onset rheumatic and musculoskeletal disease, congenital heart disease, asthma, or HIV. The barriers we identified might also contribute to the poor outcomes among young adults with other chronic conditions wherein care transitions are associated with lower likelihood of adherence to treatment, obtaining medical and specialty follow-up and satisfaction with their care, and excess hospitalization, morbidity, and mortality [61–65]. In addition, the evidence and resources developed to address these adverse outcomes could inform childhood cancer survivorship efforts. For example, recent reviews of factors that influence the care transition of young adults with chronic illness suggest essential targets for intervention to be self-management skills, trust in adult care, self-efficacy, social support, the patient’s gender and social position, the trust between child and adult carers, and interdisciplinary cooperation [46, 66, 67]. Existing guidelines might also be relevant, such as the 2011 clinical algorithm developed by the American Academy of Pediatrics, the American Academy of Family Physicians, and the American College of Physicians to ensure that high-quality, developmentally appropriate healthcare services are available in an uninterrupted manner, a well-timed transition is specific to each person, and coordination of patient, family, and provider responsibilities enable young adults to optimize their ability to assume adult roles and activities [68]. Similar to service development for young adults with various chronic conditions, incorporating CCS perspectives into healthcare service development and delivery is critical to the goal of patient-centered care and key to accessible, acceptable, appropriate, and effective healthcare.
